# Thank You to All Our Reviewers in 2023

**DOI:** 10.1055/s-0044-1786524

**Published:** 2024-04-29

The editorial board of Avicenna Journal of Medicine would like to thank the following reviewers who have reviewed manuscripts for the journal in 2023:

Aula Abbara

Ghadir Abbas

Kenneth Abbott

Maram Abdaljaleel

Saud Abdullah

Muhammmed Adeniyi

Ashfaque Ahmad

Aya Al Habbal

Abdulrahman Al Halak

Moamen Al Zoubi

Samer Al-Khudari

Aghiad Al-Kutoubi

Ghiath Alahamad

Fares Alahdab

Ghaith Alfakhry

Moath Said Alfawara

Bahaa Alhaffar

Eman Abid Fahad Alhasnawi

Hamid Ali

Munzer Alkhalil

Eyad Almallouhi

Ahmed Alnajar

Suliman Alradawi

Abdul Hamid Alraiyes

Mohammad Alsakkal

Muhammad Alsayid

Aya Alsharif

Anas Alshawa

Ertuğrul Altınbilek

Tareq Alyousef

Opada Alzohaili

Muayad Alzuabi

Adam Anderson

Mustafa Arslan

Richard Atkins

Joseph Attallah

Hrayr Attarian

Muhammad Eyad BaAth

Mohamad Badawi

Walid Ballourah

Cihan Bedel

Fadi Bouri

Abdelkader Chaar

Hassan Chamsi-Pasha

Arjun Chatterjee

Ammar Darwish

Jimoh Nafisat Dasola

Surender Deora

Anju Dinkar

Moutaz El Kadri

Orwa Elaiwy

Sana Farooki

Martin O Furr

Maarouf Gorra Al Nafouri

Tamim Hamdi

M Bakri Hammami

Mohammad Khalid Hamza

Ibrahem Hanafi

Uma Hariharan

Bashar Hasan

Taichi J Hinata

Ahmad Iftikhar

Juman Isstaif

Ahmad Kaako

Yassar Kanawati

Said Khayyata

Kheder Kheder

Ekin Kırcalı

Ahmad Mansour

Katherine Mitsouras

Isam Moghamis

Ahmed Ashraf Mohamed

Homam Moussa Pacha

Emir Muzurović

Naif Alhawiti

Hala Nas

Marwa Niazy

Ahmed Abd Al-Wahab Nugud

Fatima Obeidat

Erol Öten

Majed Pharaon

Jamir Pitton Rissardo

Bana Sabbagh

Abdulghani Sankari

Bisher Sawaf

Gabriel Schroeder

Paula R Seffens

Mohammed Nader Shalaby

Saurabh RamBihariLal Shrivastava

Annie Sparrow

Murgesan Subramaniam

Salim Surani

Abdul Rahman Tarabishy

Aladily Tariq

Rahul Thakur

Abdulhakim Tlimat

Anand Raj Vaithy

Vishal Vennu

Paul L Weygandt

Mohammad Yousef

Obada Zayegh

